# Peyronie’s disease in Spain: a prospective study

**DOI:** 10.1093/sexmed/qfag006

**Published:** 2026-05-11

**Authors:** François Peinado-Ibarra, Borja García-Gómez, Manuel Alonso-Isa, Carlos Simón-Rodríguez, Juan García-Cardoso, Josep Torremadé-Barreda, Félix Campos-Juanatey, Rodrigo García-Baquero, Mario Álvarez-Maestro, Esaú Fernández-Pascual, Juan Ignacio Martínez-Salamanca, José Martínez-Jabaloyas, Mariano Rosselló-Gayá, Venancio Chantada, Francisco Cáceres-Rodríguez, Joaquim Sarquella, Eduard Ruiz-Castañé, Enrique Lledó, Manuel Fernández-Arjona, Luis Fiter, José Ramón Cortiñas, Javier Romero-Otero

**Affiliations:** Urology Department, Hospital Universitario Quirón Ruber Juan Bravo 39, Universidad Europea de Madrid, Facultad de Medicina, 28006 Madrid, Spain; Urology Department, Hospital Universitario 12 de Octubre, 28041 Madrid, Spain; Urology Department, Hospital Universitario 12 de Octubre, 28041 Madrid, Spain; Urology Department, Hospital Universitario Fundación Jiménez Díaz, 28040 Madrid, Spain; Urology Department, Hospital Universitario Fundación Jiménez Díaz, 28040 Madrid, Spain; Urology Department, Hospital Clínic de Barcelona, 08036 Barcelona, Spain; Urology Department, Hospital Universitario Marqués de Valdecilla, 39008 Santander, Spain; Urology Department, Hospital Universitario Puerta del Mar, 11009 Cádiz, Spain; Urology Department, Hospital Universitario La Paz, 28046 Madrid, Spain; Urology Department, LYX Instituto de Urología, Universidad Francisco de Vitoria, 28006 Madrid, Spain; Urology Department, LYX Instituto de Urología, Universidad Francisco de Vitoria, 28006 Madrid, Spain; Urology Department, Hospital Clínico Universitario de Valencia, 46010 Valencia, Spain; Urology Department, Centro de Urología Andrología y Medicina Sexual, 07010 Palma, Illes Balears, Spain; Urology Department, Hospital Universitario de A Coruña (CHUAC), 15006 A Coruña, Spain; Urology Department, Hospital Universitario de Álava, 01009 Vitoria, Spain; Urology Department, Fundación Puigvert, 08025 Barcelona, Spain; Urology Department, Fundación Puigvert, 08025 Barcelona, Spain; Urology Department, Hospital General Universitario Gregorio Marañón, 28007 Madrid, Spain; Urology Department, Hospital Universitario del Henares, 28822 Coslada, Madrid, Spain; Urology Department, Hospital Universitario Severo Ochoa, 28914 Leganés, Madrid, Spain; Urology Department, Hospital Universitario Río Hortega, 47012 Valladolid, Spain; Urology Department, Hospital Universitario 12 de Octubre, 28041 Madrid, Spain

**Keywords:** epidemiology, penile curvature, Peyronie’s disease, Peyronie’s disease questionnaire (PDQ)

## Abstract

**Background:**

Peyronie’s disease (PD) research has limitations due to heterogeneity in time of presentation, severity, deformity characteristics, and associated conditions.

**Aim:**

To provide further insight into clinical characteristics of patients, PD treatments and patient perceptions of outcomes in clinical practice in Spain.

**Methods:**

A total of 601 men from 15 Spanish andrology centers were included in this observational, prospective multicenter cohort study. Enrolled patients (aged ≥18 years with confirmed PD diagnosis, naïve to specific therapy and without congenital penile curvature) were evaluated by detailed medical history and physical examination including plaque characteristics and curvature. Sexual function was assessed by the International Index of Erectile Function-5 and the Peyronie’s Disease Questionnaire. The characteristics of treatment used for PD as well as patient subjective outcomes were analyzed in cases involving curvature assessments.

**Outcomes:**

The primary outcome was demographic and clinical characterization of PD patients; secondary outcomes included PD treatment used in clinical practice and patient satisfaction.

**Results:**

The median age (interquartile range, IQR) was 57 (49.0-63.0) years with a median duration of PD of 12 (6.0-18.0) months. There were 246 (41.6%) patients in the acute phase of the disease. The most common nonsurgical treatments were traction therapy (36.0%), collagenase injections (29.3%), and daily tadalafil (22.7%). The most frequently used surgical technique was penile plication (62.3%) followed by grafting and plaque incision (34.0%). The median Peyronie’s Disease Questionnaire and International Index of Erectile Function-5 scores were 19.0 (12.0-28.0) and 18.0 (13.0-22.5), respectively. Curvature assessment was performed in 331 patients, using the Kelami test in 316 (96.0%) or intracavernous injection of a vasoactive agent in 143 (45.5%).

**Clinical implications:**

Many patients are misdiagnosed, and treatment may take a long time; understanding the profile of PD, as well as satisfaction with real-life treatment outcomes, can aid clinicians in selecting more appropriate treatments.

**Strengths and Limitations:**

As a free collaborative registry, not all patient data were collected, and therefore not all data were available throughout the study, and a longer follow-up period would have given us more information to compare the pretreatment and post-treatment situations. However, this study provides a description of clinical characteristics of a large real-world cohort of men with PD from a considerable number of sites nationwide.

**Conclusions:**

We found a wide variety of treatments used in clinical practice, including those with poor evidence of efficacy, which suggests the need for a better understanding of PD for developing the most effective treatment protocol.

## Introduction

Peyronie’s disease (PD) is a fibrotic condition of the penile tunica albuginea leading to plaque formation, penile deformation, penile pain, and in some patients, erectile dysfunction (ED).^1,2^ Psychologically, PD can also lead to feelings of shame, inadequacy, reduced self-worth, insecurity, dysmorphia, isolation, anger, and depression.^3^ The disease is characterized by an initial or acute inflammatory phase usually lasting 5-7 months, although it can last up to 12 months or even longer, accompanied by painful erections and a changeable penile deformity. The stable or chronic phase begins when the acute phase subsides and it is characterized by a lack of symptom changes and pain improvement/resolution.^1^ The etiology of PD is multifactorial and not fully understood, and consequently patients are often misdiagnosed (eg, with ED), and time to diagnosis and treatment may be long. The most widespread etiological theory implicates penile trauma, either as an acute traumatic event or as repetitive microtrauma that may occur during sexual intercourse.^4^ The initial evaluation should include medical and sexual history, symptoms, and identification of penile plaque by palpation.^5^ Imaging can also be used to identify the site and characteristics of the plaque.^6^

The spectrum of PD treatment includes appropriate counseling, medical therapy, and minimally invasive and surgical treatment options. Therapeutic goals should be tailored to the patient’s preferences and disease characteristics. Thus, in patients with minimal bother, observation alone may be sufficient, while extracorporeal shock wave treatment and penile traction may only be effective for treating penile pain and penile deformity, respectively. Similarly, surgical straightening with tunica albuginea shortening or lengthening techniques may be performed only in the chronic phase so as to prevent recurrence.^5,7–9^ Surgery can result in penile shortening, ED, glans hypoesthesia, residual curvature, and unsightly suture knots beneath the skin.^10^ Therefore, expected outcomes and risks of possible interventions should be discussed with patients.

Despite the availability of numerous treatment options, the lack of consensus and heterogeneity among guidelines recommendations result in uncertainty for many clinicians facing PD in clinical practice. An optimal treatment algorithm applying a multimodal approach is yet to be defined. Additionally, available research on PD is limited due to heterogeneity in time of presentation, severity, characteristics of the deformity, and associated conditions.

This real-world study sought to provide further insight into clinical characteristics and therapeutic management of patients with PD and their perceptions of outcomes in the real-world setting in Spain.

## Methods

### Study design and participants

This was a prospective, multicenter observational (patient registry) cohort study conducted in 15 Spanish andrology centers according to the Declaration of Helsinki and national regulations. It was approved by the ethics committee of Hospital 12 de Octubre (Madrid, Spain) and patients gave their written informed consent.

Eligible patients were consecutive men aged ≥18 years with a confirmed diagnosis of PD and who were naïve to specific therapy (ie, surgical and/or nonsurgical). Patients with congenital penile curvature were excluded. Enrolled patients were evaluated by detailed medical history, including demographics, duration of disease, previous studies of ED or endoscopic surgeries, presence of subjective pain, shortening, narrowing/hourglass deformity, and by physical examination including plaque characteristics (location, size, and consistency) and curvature (direction and angle). Stretched penile length was measured from the pubis to the tip of the glans (meatus) on the dorsal aspect of the penis with maximal extension in the flaccid state. Sexual function was assessed by the International Index of Erectile Function (IIEF-5)^11^ and by the Peyronie’s Disease Questionnaire (PDQ).^12^

The perioperative data recorded included type of surgery, type of anesthesia, operative time, residual curvature, need of additional plication procedures, use of urethral catheter, bandage time, and postoperative management and complications.

To evaluate subjective post-treatment outcomes, a nonvalidated ad hoc 9-item questionnaire including simple questions was designed (Supplementary Material S1).

The treating physicians determined the treatment (ie, surgical, and/or nonsurgical) according to the patient’s individualized need for medical care. Patients were tracked longitudinally, and subsequent interventions and outcomes were recorded.

The primary endpoint was the demographic and clinical characterization of PD patients. Secondary endpoints included characteristics of PD treatments used under routine clinical practice conditionsand patient satisfaction with treatment.

### Statistical analysis

A descriptive statistical analysis was performed on the study variables estimating the central tendency and dispersion (mean ± standard deviation [SD], median, and interquartile range [IQR]) for quantitative variables and counts and percentages for qualitative variables. Statistical analyses were performed using SAS version 9.4 (SAS Institute Inc., Cary, NC, USA).

## Results

### Patient characteristics

Between January 2019 and September 2019, a total of 601 men, with a median (IQR) age of 57.0 (49.0-63.0) years, were included in the study. The most common comorbidities were hypercholesterolemia (30.0%), hypertension (27.1%), and diabetes (15.0%). Overall, 3.3% of patients presented with Dupuytren’s disease (DD). The baseline characteristics of patients are described in Table 1.

**Table 1:** Patient baseline characteristics.

Patient characteristics	*N* = 601
**Age** (years) median (IQR)	57.0 (49.0-63.0)
**BMI** (kg/cm^2^) median (IQR)	26.9 (24.5-29.0)
**Smoking**, *n* (%)	129 (21.5)
**Comorbidities**, *n* (%)	
Hypertension	163 (27.1)
Diabetes mellitus	90 (15.0)
Hypercholesterolemia	180 (30.0)
Connective tissue disease	14 (2.3)
Dupuytren’s disease	20 (3.3)
**Previous circumcision**, *n* (%)	35 (5.8)
**Previous prostate TUR**, *n* (%)	23 (3.8)
**Intracavernosal injections**, *n* (%)	28 (4.7)
**Duration of PD** (months) median (IQR)	12.0 (6.0-18.0)
**Painful phase**, *n* (%)	246 (41.6)
Duration (months) median (IQR)	6.0 (4.0-10.0)
**IIEF-5** ^a^ (points) median (IQR)	18.0 (13.0-22.5)
**PDQ** ^b^ (points) median (IQR)	19.0 (12.0-28.0)
PDQ-PS (0-24)	10.0 (6.0-14.0)
PDQ-PP (0-30)	5.0 (3.0-7.0)
PDQ-SB (0-20)	2.0 (1.0-2.0)
**Physical examination**	
Palpable plaque, *n* (%)	486 (82.1)
Dorsal location, *n* (%)	377 (77.6)
Ventral location, *n* (%)	20 (4.1)
Lateral location, *n* (%)	40 (8.2)
Stretched penile length^c^ (cm) median (IQR)	13.0 (10.0-14.5)

Abbreviations: IIEF-5, 5-item version of International Index of Erectile Function; IQR, interquartile range; PDQ, Peyronie’s Disease Questionnaire; PDQ-PP, PDQ-Penile Pain; PDQ-PS, PDQ-Psychological, and Physical Symptoms; PDQ-SB, PDQ-Symptom Bother; TUR, transurethral resection.

^a^IIEF-5 score derived from available data from 416 patients.

^b^PDQ score derived from available data from 215 patients who had experienced vaginal penetration in the last 3 months.

^c^Penile length was determined by measurement of the stretched dorsal length of the penis from the pubis to the urethral meatus.

The median duration of PD was 12.0 (6.0-18.0) months. Overall, 41.6% of patients were in the acute or painful phase of the disease, whose median duration was 6.0 months. PDQ responses were obtained from 248 (41.3%) men who had engaged in vaginal intercourse within the previous three months. With a median PDQ score of 19.0 (12.0-28.0), the highest score was obtained in the psychological and physical symptoms scale. The median IIEF-5 score was 18.0 (13.0-22.5). The responses obtained for PDQ and IIEF-5 items are shown in Tables S1 and S2, respectively.

Plaque was palpable in 486 (82.1%) patients, in 77.6% arising in the dorsal aspect of the penis. The median stretched penile length was 13.0 (10.0-14.5) cm.

Curvature assessment was performed in 331 patients. The location and degree of curvature were evaluated using self-photographs of the penis at maximum erection, from above, laterally, and frontally (Kelami test)^13^ in 96.0% of patients. Direction of penile curvature was dorsal in all patients and of these, it was lateral in 34.9%, with an angle of 30-60° in 57.4% and 48.7%, respectively. Overall, 9.2% patients had hourglass deformity (Table 2).

**Table 2:** Penile curvature parameters and ultrasound measurements.

Parameters	*N* = 331
**Curvature assessment methodology**, *n* (%)	
Kelami test	316 (96.0)
Intracavernous prostaglandin injection test	143 (45.5)
**Curvature direction and degree**, n (%)	
Dorsal	331 (100.0)
<30°	55 (16.6)
30-60°	190 (57.4)
60-90°	70 (21.1)
>90°	16 (4.8)
Lateral	114 (34.9)
<30°	42 (37.2)
30-60°	55 (48.7)
60-90°	13 (11.5)
>90°	3 (2.7)
**Hourglass**, *n* (%)	30 (9.2)
**US imaging assessment** ^a^ ^,^ ^b^, *n* (%)	94 (53.7)
**Plaque on US**, *n* (%)	65 (69.1)
Calcification, *n* (%)	35 (53.8)
Major plaque size (mm) mean (SD)	14.4 (10.2)
**Cavernous septum involvement**, *n* (%)	25 (28.1)
**Erectile function** (cm/s) median (IQR)	
Peak systolic velocity	14.0 (11.0-15.0)
End diastolic velocity	2.0 (2.0-4.0)

Abbreviations: IQR, interquartile range; US, ultrasound.

^a^After intracavernosal injection.

^b^Penile Duplex-Doppler ultrasound was performed.

Ultrasound was used to examine penile plaques in 94 patients. Calcification was detected in 35 of the 65 (53.8%) patients with confirmed plaques. The mean (SD) size of the major plaque was 14.4 (10.2) mm. Involvement of the cavernous septum was detected in 25 (28.1%) patients.

### Treatment strategies

Overall, 82 (25.2%) patients underwent surgery, 183 (56.3%) received nonsurgical treatments, 23 (7.1%) underwent surgery and received nonsurgical treatments, and 37 (11.4%) were nontreated (Table 3).

**Table 3:** Management of patients with Peyronie’s disease.

Management	*N* = 325 *n* (%)
**Only surgical**	82 (25.2)
**Only nonsurgical**	183 (56.3)
**Surgical + nonsurgical**	23 (7.1)
**Type of treatment** ^a^	
Traction therapy	119 (36.0)
Surgery	105 (31.7)
Intralesional therapy	
CCH	97 (29.3)
Verapamil	5 (1.5)
Corticoids	1 (0.3)
Oral therapy	
Daily tadalafil	75 (22.7)
Pentoxifylline	44 (13.3)
Vitamin E	13 (3.9)
l-Arginine	8 (2.4)
Colchicine	1 (0.3)
NSAID (pain relief)	3 (0.9)
ESWT	3 (0.9)
Vacuum therapy	1 (0.3)
**Nontreated**	37 (11.4)

Abbreviations: CCH, collagenase *Clostridium histolyticum*; ESWT, extracorporeal shockwave therapy; NSAID, nonsteroidal anti-inflammatory drugs.

^a^Multiple response. Can add up to more than 100%.

The most common nonsurgical treatments were traction therapy (36.0%), collagenase injections (29.3%), daily tadalafil (22.7%), and pentoxifylline (13.3%).

The median time from onset of PD to surgery was 18.0 months. Among the 105 patients who underwent surgery, data about the performance of penile plication, plaque incision, plaque excision, and penile prosthesis was available, in 53, 53, 51, and 58 patients, respectively. The preferred surgical technique was penile plication (33/53; 62.3%), with Nesbit penile plication being the predominant technique (19/53, 35.8%), followed by grafting and plaque incision (18/53, 34.0%). After plaque incision or excision, the graft materials of choice included collagen fleece (17/53; 32.1%), intestinal submucosa (4/53; 7.5%), and pericardium (6/52; 11.3%).

During surgery, general anesthesia was most commonly used (50.0%). Complete curvature correction (residual curvature ≤15°) was achieved in 47 (47/59, 80.0%) patients and median penile length before and after surgery was 12.6 (12.0-14.0) and 12.3 (12.0-14.0) cm, respectively. About half of patients required penile rehabilitation after surgery. Postoperative complications were noted in 8 (12.7%) patients (Table 4).

**Table 4:** Perioperative data.

Parameter	N	Value
**Time from onset of disease to surgery** (months) median (IQR)	62	18.0 (14.0-22.0)
**Preoperative length of stretched flaccid penis** (cm) median (IQR)	35	12.6 (1.2-14.0)
**Curvature degree**, median (IQR)	58	62.5 (45.0-90.0)
**Type of anaesthesia**, *n* (%)	62	
Local		7 (11.3)
Regional		24 (38.7)
General		31 (50.0)
**Type of surgery**, n (%)		
Penile plication^a^	53	33 (62.3)
Grafting^b^	53	24 (45.3)
Plaque incision	53	18 (34.0)
Plaque excision	51	6 (11.8)
Inflatable penile prosthesis	58	10 (17.2)
**Operative time** (min) mean (SD)	58	90.8 (42.9)
**Urethral catheter**, *n* (%)	63	42 (66.7)
**Postoperative length of penis (after surgery)** (cm) median (IQR)	30	12.3 (12.0-14.0)
**Residual curvature**, *n* (%)	59	7 (11.9)
**Additional plication**, *n* (%)	63	8 (12.7)
**Bandage time** (h) mean (SD)	63	50.6 (31.9)
**Postoperative management of patients**, *n* (%)		
Antibiotics	62	33 (53.2)
Penile rehabilitation^c^	61	30 (49.2)
**Complications**, *n* (%)	63	8 (12.7)
Hematoma		5 (7.9)
Wound infection		1 (1.6)
Oedema/ecchymosis		1 (1.6)
**Hospital stay** (days) mean (SD)	63	23.3 (17.4)

Abbreviations: IQR, interquartile range; N, patients with available data; NA, not available SD, standard deviation.

^a^Includes the following techniques: Yachia (*n* = 4, 7.5%), Nesbit (*n* = 19, 35.8%), Essed/Schroeder (*n* = 3, 5.7%), 16-dot (*n* = 2, 3.8%), 6-dot repair with Ticron sutures (*n* = 1, 1.9%), NA (*n* = 1, 1.9%), and combined Nesbit and 16-dots procedures (*n* = 1, 1.9%).

^b^Grafting materials used were collagen fleece (*n* = 17, 32.1%), intestinal submucosa (*n* = 4, 7.5%), pericardium (*n* = 6, 11.3%), and not available (*n* = 1, 1.9%).

^c^Includes vacuum therapy (*n* = 2, 6.7%), penile extender (*n* = 18, 60.0%), daily tadalafil (*n* = 10, 33.3%), manual stretching (*n* = 22, 73.3%), and infrapubical early implant activation (*n* = 1, 3.3%).

Overall, 28.7% (25/87) of patients had long-term complications related to the treatment (ie, surgical and nonsurgical), the most common being ecchymosis or edema in 32.0% (8/25).

### Patient subjective outcomes

Table 5 shows that, overall, 42 (56.7%) patients reported that they currently had a straight or almost straight penis. Although penile shortening was reported by 28 (46.7%) patients, this was ≥2 cm in only 4 (6.7%). A higher proportion of surgical patients (66.7%) achieved greater overall resolution of curvature compared to nonsurgical treatments (27.3%).

**Table 5:** Patient subjective outcomes after treatment.

Outcomes	Total *N* = 90		Surgery *N* = 7		Nonsurgery *N* = 44	
	*m*	*n* (%)	*m*	*n* (%)	*m*	*n* (%)
**Patient perceived penile curvature**	74		6		33	
Completely correct		20 (27.0)		1 (16.7)		2 (6.1)
Almost completely correct		22 (29.7)		3 (50.0)		7 (21.2)
Partially correct		21 (28.4)		1 (16.7)		14 (42.4)
Similar to before		11 (14.9)		1 (16.7)		10 (30.3)
**Patient perceived penile shortening**	60		4		29	
No		32 (53.3)		2 (50.0)		16 (55.2)
Present but less than 2 cm		24 (40.0)		2 (50.0)		11 (37.9)
Greater than 2 cm		4 (6.7)		-		2 (6.9)
**Reduction of penile sensitivity**	63	7 (11.9)	5	1 (20.0)	27	2 (7.4)
**Ejaculation and orgasm disorders**	59	7 (11.9)	5	1 (20.0)	23	4 (17.4)
**Satisfactory sexual relations**	74	43 (58.1)	6	4 (66.7)	33	16 (48.5)
**Treatment of erectile dysfunction**	67	25 (37.3)	6	3 (50.0)	30	10 (33.3)
**Degree of satisfaction with treatment**	71		7		28	
Very satisfied		17 (23.9)		2 (28.6)		3 (10.7)
Somewhat satisfied		35 (49.3)		3 (42.9)		14 (50.0)
Neither satisfied nor dissatisfied		9 (12.7)		1 (14.3)		5 (17.9)
Very dissatisfied		10 (14.1)		1 (14.3)		6 (21.4)
**The patient would recommend the same treatment**	56	50 (89.3)	6	5 (83.3)	21	17 (81.0)
**Degree of improvement of clinical condition**	27		3		10	
Large improvement		13 (48.1)		2 (66.7)		1 (10.0)
Moderate improvement		9 (33.3)		-		4 (40.0)
Similar to before		4 (14.8)		-		4 (40.0)
No improvement		1 (3.7)		1 (33.3)		1 (10.0)

*m*, number of patients with available data; *N*, number of patients with curvature assessment; *n*, patients with event.

Overall, 58.1% of patients reported satisfactory sexual relations, with this percentage being higher among surgical (66.7%) than nonsurgical (48.5%) patients. Patients who underwent surgery were more satisfied with their experience (satisfied/very satisfied: 71.5% in surgical and 60.7% in nonsurgical patients). Most patients (89.3%) would choose the same treatment.

## Discussion

This study describes the clinical characteristics of a Spanish cohort of 601 men diagnosed with PD. This is therefore the largest cohort of PD patients described in Spain to our knowledge. The median age of patients was 57 years with a median duration of PD of 12 months. We found that almost half (42%) of the patients were in the active phase during the initial visit. These results support that PD prevalence is higher for older men, with a typical age of onset of 50-60 years, as aging may contribute to the loss of stiffness in the penis, making it more vulnerable to microtrauma and fibrous plaque formation.^1,14^ Hypercholesterolemia, hypertension, and diabetes were the most common comorbidities. Several studies have already reported an association between PD and certain risk factors.^15–18^ Thus, hypertension or hyperlipidemia and diabetes can cause abnormal wound healing, and exacerbate fibrotic processes in erectile tissue, which can increase the risk of PD.^19,20^ DD occurred in only 3% of patients. Indeed, although some studies have noted the co-occurrence of PD with DD,^21,22^ the prevalence of coexisting DD among PD patients varies from 0.01% to 58.8%.^15,17,21–25^ Peyronie’s plaques were detected in 82% of the males studied, mainly on the dorsal aspect of the penis (78%), in line with results of previous studies.^26^

Our results support the significant psychosocial impacts of PD on men based on PDQ scores, which are largely related to relationship problems and a loss of penile length.^27^ Thus, patients indicated that their most bothersome or distressing symptoms were penis appearance (eg, bend or curvature of the penis), changes and frequency of vaginal intercourse, as well as difficulties with certain positions for intercourse. A penile bend or curvature can make it difficult or restrict patients from engaging in sexual intercourse, potentially leading to reduced sexual activity.^28,29^ Since PD has an important impact on a patient’s sexual life and self-perception, it is important to ask about these experiences during the initial evaluation of the disease. This will allow the physician to better assess the patient’s motivation for treatment. We also found that men with PD showed mild ED based on the IIEF-5 score. Penile curvature was mainly evaluated with the Kelami method (96%). As opposed to congenital penile curvature, in which ventral curvature dominates, PD may have curvatures in either direction, and may be uni- or biplanar.^30^ PD-related penile curvature is most commonly dorsal,^1^ as confirmed in all patients whose curvatures were examined in our cohort. Only 9% of the cases had an hourglass deformity. Ventral curvature cases would probably have been found if curvature assessments had been conducted in the total population.

The observed treatment patterns for patients with PD include surgical and nonsurgical procedures, including oral, topical, intralesional, extracorporeal shockwave, and traction therapy. Although there have been many treatments suggested and used in treating PD, only collagenase *Clostridium histolyticum* (CCH) is approved by the Food and Drug Administration (FDA) and the European Medicines Agency (EMA) for the treatment of PD. As such, physicians must determine nonsurgical treatment options according to limited placebo-controlled clinical trials.^8,31^ Since oral therapy has not proven to be effective and is associated with potential side effects and costs, it is not currently recommended. Our results contrast with these recommendations, where the use of vitamin E, colchicine, or l-Arginine is indeed minimal, but tadalafil and pentoxifylline were used in 23% and 13% of cases, respectively. This could be explained by the fact that many patients choose noninvasive treatment, along with a lack of updating of physicians on guidelines, as the only randomized placebo-controlled trial on pentoxifylline has been withdrawn.^8,32,33^ There is still a need to define an optimal treatment algorithm using a multimodal approach, making it difficult to provide recommendations in an everyday, real-world setting. Stem-cell therapy is an emerging field that aims to develop new strategies to repair or replicate tissues and organs, but clinical trials are still pending, and it should only be used in clinical research settings.^34^

Surgical management of the disease is usually kept for later stages of the disease after it has stabilized. Some authors suggest a surgical intervention at a 6-12-month period.^35^ The chronic stage has been shown to occur 6-12 months after the acute phase, where pain disappears and the deformity stabilizes^36^. We found that in clinical practice, this procedure can be performed even later, up to 18 months (median) after onset of symptoms.

The most common surgical treatment for PD is tunical plication,^37^ and the current analysis supports this observation. No serious postoperative complications were reported.

Rehabilitation after surgery is critical in reducing the risk of postoperative ED, length loss and to promote a smooth recovery. Patients are usually seen 2 weeks after surgery, at which point stretch therapy and massage are introduced.^38,39^ We found, however, that less than half of the patients underwent this postoperative rehabilitation.

The correction of curvature can enhance quality of life and sexual function. The use of intraplaque injections, particularly CCH, have shown strong evidence of beneficial outcomes in PD. Thus, the IMPRESS I/II trials^40^ showed significant improvements in curvature with collagenase injections. However, CCH may not be suitable for all patients. Success rates of CCH vary and may be less effective in comparison to the available surgical options for some patients, with surgery providing higher rates of successful curvature correction.^41^ This could explain why surgical patients showed a higher observed rate of fully corrected curvature compared to that perceived by the entire cohort (88% vs. 57%). Surgery, however, may cause further loss of penile length.^27^

Despite decreased penile length (47%) and ED (37%), patients reported high satisfaction as nearly 90% stated they would repeat the treatment received, and more than half (58%) said they were satisfied with their present sexual life.

The study has some limitations that should be acknowledged. First, being a free collaborative registry, which uses information recorded for non-research purposes in the medical chart, with not all patient data collected, and therefore not all data were available throughout the study. Second, due to the short study duration, not all patients were followed up. A longer follow-up period would have given us more information to compare the pretreatment and post-treatment situations. Comparisons between surgical and nonsurgical patients are based on small figures and should be interpreted with caution. Nonetheless, this study provides a description of clinical characteristics of a large real-world cohort of men with PD from a considerable number of sites nationwide.

In order to assess the risk of PD and develop both long- and short-term strategies for health maintenance, it is important to be aware of the factors underlying PD, how PD is linked to other comorbid conditions, and how treatment affects both health outcomes and patient satisfaction.

## Conclusion

We found that in real clinical practice a wide variety of treatments are used, both medically, most of which have little evidence of efficacy and are even not currently recommended, as well as surgically, which has the highest level of satisfaction. Additionally, the negative effects of PD on sexual life and self-awareness must be considered. This supports that there is a genuine need for progress in the understanding of PD for developing the best treatment protocol for these men. More clinical registries would contribute to advancing disease knowledge.

## Supplementary Material

qfag006_Supplemental_Files

## References

[1] Mulhall JP, Schiff J, Guhring P. An analysis of the natural history of Peyronie's disease. J Urol. 2006;175(6):2115–2118; discussion 2118. 10.1016/S0022-5347(06)00270-9 16697815

[2] Stuntz M, Perlaky A, des Vignes F, Kyriakides T, Glass D. The prevalence of Peyronie's disease in the United States: a population-based study. PLoS ONE. 2016;11(2):e0150157–e0150157. 10.1371/journal.pone.0150157 26907743 PMC4764365

[3] Hartzell R . Psychosexual symptoms and treatment of Peyronie's disease within a collaborative care model. Sex Med. 2014;2(4):168–177. 10.1002/sm2.45 25548648 PMC4272248

[4] Bjekic MD, Vlajinac HD, Sipetic SB, Marinkovic JM. Risk factors for Peyronie's disease: a case-control study. BJU Int. 2006;97(3):570–574. 10.1111/j.1464-410X.2006.05969.x 16469028

[5] Hatzimouratidis K, Eardley I, Giuliano F, et al. EAU guidelines on penile curvature. Eur Urol. 2012;62(3):543–552. 10.1016/j.eururo.2012.05.040 22658761

[6] Pawłowska E, Bianek-Bodzak A. Imaging modalities and clinical assessment in men affected with Peyronie's disease. Pol J Radiol. 2011;76(3):33–37. 22802839 PMC3389927

[7] Levine LA, Larsen SM. Surgery for Peyronie's disease. Asian J Androl. 2013;15(1):27–34. 10.1038/aja.2012.92 23178395 PMC3739133

[8] Teloken P, Katz D. Medical management of Peyronie's disease: review of the clinical evidence. Med Sci (Basel). 2019;7(9):96. 10.3390/medsci7090096 PMC6780399 31540526

[9] Yafi FA, Pinsky MR, Sangkum P, Hellstrom WJ. Therapeutic advances in the treatment of Peyronie's disease. Andrology. 2015;3(4):650–660. 10.1111/andr.12058 26097120

[10] Garaffa G, Trost LW, Serefoglu EC, Ralph D, Hellstrom WJG. Understanding the course of Peyronie's disease. Int J Clin Pract. 2013;67(8):781–788. 10.1111/ijcp.12129 23869679

[11] Rosen RC, Riley A, Wagner G, Osterloh IH, Kirkpatrick J, Mishra A. The international index of erectile function (IIEF): a multidimensional scale for assessment of erectile dysfunction. Urology. 1997;49(6):822–830. 10.1016/s0090-4295(97)00238-0 9187685

[12] Coyne KS, Currie BM, Thompson CL, Smith TM. Responsiveness of the Peyronie's disease questionnaire (PDQ). J Sex Med. 2015;12(4):1072–1079. 10.1111/jsm.12838 25664497

[13] Kelâmi A . Classification of congenital and acquired penile deviation. Urol Int. 1983;38(4):229–233. 10.1159/000280897 6879869

[14] Rhoden EL, Teloken C, Ting HY, et al. Prevalence of Peyronie's disease in men over 50-y-old from Southern Brazil. Int J Impot Res. 2001;13(5):291–293. 10.1038/sj.ijir.3900727 11890516

[15] Carrieri MP, Serraino D, Palmiotto F, Nucci G, Sasso F. A case-control study on risk factors for Peyronie's disease. J Clin Epidemiol. 1998;51(6):511–515. 10.1016/s0895-4356(98)00015-8 9636000

[16] Oktar T, Kendirci M, Sanli O, Kadioglu A. The impact of risk factors on the severity of penile deformity in 484 Peyronie patients. In: Levine LA (ed). Annual Meeting of the American-Urological-Association, Philadelphia PA: Lippincott Williams & Wilkins; 2003: 274–274.

[17] Perimenis P, Athanasopoulos A, Gyftopoulos K, Katsenis G, Barbalias G. Peyronie's disease: epidemiology and clinical presentation of 134 cases. Int Urol Nephrol. 2001;32(4):691–694. 10.1023/a:1014485204205 11989566

[18] Usta MF, Bivalacqua TJ, Jabren GW, et al. Relationship between the severity of penile curvature and the presence of comorbidities in men with Peyronie's disease. J Urol. 2004;171(2 Pt 1):775–779. 10.1097/01.ju.0000097498.34847.7c 14713809

[19] El-Sakka AI, Hassoba HM, Pillarisetty RJ, Dahiya R, Lue TF. Peyronie's disease is associated with an increase in transforming growth factor-beta protein expression. J Urol. 1997;158(4):1391–1394. 9302128

[20] Kendirci M, Trost L, Sikka SC, Hellstrom WJ. Diabetes mellitus is associated with severe Peyronie's disease. BJU Int. 2007;99(2):383–386. 10.1111/j.1464-410X.2007.06611.x 17313425

[21] Nugteren HM, Nijman JM, de Jong IJ, van Driel MF. The association between Peyronie's and Dupuytren's disease. Int J Impot Res. 2011;23(4):142–145. 10.1038/ijir.2011.18 21633367

[22] Rhoden EL, Riedner CE, Fuchs SC, Ribeiro EP, Halmenschlager G. A cross-sectional study for the analysis of clinical, sexual and laboratory conditions associated to Peyronie's disease. J Sex Med. 2010;7(4 Pt 1):1529–1537. 10.1111/j.1743-6109.2009.01584.x 19912489

[23] Ralph DJ, Schwartz G, Moore W, Pryor JP, Ebringer A, Bottazzo GF. The genetic and bacteriological aspects of Peyronie's disease. J Urol. 1997;157(1):291–294. 8976282

[24] Chilton CP, Castle WM, Westwood CA, Pryor JP. Factors associated in the aetiology of Peyronie's disease. Br J Urol. 1982;54(6):748–750. 10.1111/j.1464-410x.1982.tb13640.x 7150935

[25] Deveci S, Hopps CV, O'Brien K, Parker M, Guhring P, Mulhall JP. Defining the clinical characteristics of Peyronie's disease in young men. J Sex Med. 2007;4(2):485–490. 10.1111/j.1743-6109.2006.00344.x 17081219

[26] Kalokairinou K, Konstantinidis C, Domazou M, Kalogeropoulos T, Kosmidis P, Gekas A. US imaging in Peyronie's disease. J Clin Imaging Sci. 2012;2:63. 10.4103/2156-7514.103053 23230545 PMC3515929

[27] Smith JF, Walsh TJ, Conti SL, Turek P, Lue T. Risk factors for emotional and relationship problems in Peyronie's disease. J Sex Med. 2008;5(9):2179–2184. 10.1111/j.1743-6109.2008.00949.x 18638001 PMC2881678

[28] Rosen R, Catania J, Lue T, et al. Impact of Peyronie's disease on sexual and psychosocial functioning: qualitative findings in patients and controls. J Sex Med. 2008;5(8):1977–1984. 10.1111/j.1743-6109.2008.00883.x 18564146

[29] Walsh TJ, Hotaling JM, Lue TF, Smith JF. How curved is too curved? The severity of penile deformity may predict sexual disability among men with Peyronie's disease. Int J Impot Res. 2013;25(3):109–112. 10.1038/ijir.2012.48 23344164

[30] Bilgutay AN, Pastuszak AW. Peyronie's disease: a review of etiology, diagnosis, and management. Curr Sex Health Rep. 2015;7(2):117–131. 10.1007/s11930-015-0045-y 26279643 PMC4535719

[31] Tsambarlis P, Levine LA. Nonsurgical management of Peyronie's disease. Nat Rev Urol. 2019;16(3):172–186. 10.1038/s41585-018-0117-7 30397330

[32] Capoccia E, Ziegelmann M, Emmerson J, Lankford J, Ofori-Marfoh C, Levine L. Long-term patient-reported outcomes in men with Peyronie's disease undergoing nonsurgical and nonintralesional injection management. Int J Impot Res. 2021;33(1):75–81. 10.1038/s41443-020-0231-y 31988423

[33] Bole R, Ziegelmann M, Avant R, et al. MP56-16 patient's choice of health information and treatment modality for Peyronie's disease. J Urol. 2017;197(4S):e760–e761. doi:10.1016/j.juro.2017.02.1769 29795531

[34] Pozzi E, Muneer A, Sangster P, et al. Stem-cell regenerative medicine as applied to the penis. Curr Opin Urol. 2019;29(4):443–449. 10.1097/MOU.0000000000000636 31008782

[35] Langston JP, Carson CC 3rd. Peyronie disease: plication or grafting. Urol Clin North Am. 2011;38(2):207–216. 10.1016/j.ucl.2011.03.001 21621087

[36] Hussein AA, Alwaal A, Lue TF. All about Peyronie's disease. Asian J Urol. 2015;2(2):70–78. 10.1016/j.ajur.2015.04.019 29264123 PMC5730743

[37] Nehra A, Alterowitz R, Culkin DJ, et al. Peyronie's disease: AUA guideline. J Urol. 2015;194(3):745–753. 10.1016/j.juro.2015.05.098 26066402 PMC5027990

[38] Horton CE, Sadove RC, Devine CJ Jr. Peyronie's disease. Ann Plast Surg. 1987;18(2):122–127. 10.1097/00000637-198702000-00005 3551750

[39] Moncada-Iribarren I JJ, Martinez-Salamanca JI, Cabello R, Hernandez C. Managing penile shortening after Peyronie's disease surgery. McVary KT (ed). Presented at Proceedings of Annual Meeting of the American Urological Association; 2007; Anaheim, CA. Philadelphia, PA: Lippincott Williams & Wilkins; 2007.

[40] Goldstein I, Lipshultz LI, McLane M, et al. Long-term safety and curvature deformity characterization in patients previously treated with collagenase Clostridium histolyticum for Peyronie’s disease. J Urol. 2020;203(6):1191–1197. doi:10.1097/JU.0000000000000743 31922462

[41] Yafi FA, Hatzichristodoulou G, Knoedler CJ, Trost LW, Sikka SC, Hellstrom WJ. Comparative analysis of tunical plication vs. intralesional injection therapy for ventral Peyronie's disease. J Sex Med. 2015;12(12):2492–2498. 10.1111/jsm.13072 26646187

