# Supplementary material for: Peyronie’s disease in Spain: a prospective study
Source: Sex Med. 2026 May 11;14(4):qfag006. doi: 10.1093/sexmed/qfag006 (PMC13158231; doi:10.1093/sexmed/qfag006)
Supplement: qfag006_Supplemental_Files [file qfag006_supplemental_files.zip › Patient_perceived_outcomes_after_treatment_qfag006.pdf]

Patient perceived outcomes after treatment

1. Perceived curvature
    - Completely correct
    - Almost completely correct
    - Partially correct
    - Similar to before
    - Worse tan before

2. Perceived penile shortening: (Yes / No) _____; _______cm

3. Reduction of penile sensitivity

    - No
    - Yes

4. Ejaculation and orgasm disorders

    - No
    - Yes

5. Satisfactory sexual relations

    - No
    - Yes

6. Treatment for erectile dysfunction

    - Yes
    - No

7. Type of treatment used for erectile dysfunction

    - Phosphodiesterase type 5 (PDE5) inhibitors
    - Topical alprostadil
    - Intraurethral alprostadil
    - Intracavernosal injection
    - Vacuum pump

7. Perceived degree of improvement in clinical situation

    - Large improvement
    - Moderate improvement
    - Similar to before
    - Slight worsening
    - Significant worsening

8. Degree of satisfaction with treatment

    - Very satisfied
    - Somewhat satisfied
    - Neither satisfied nor dissatisfied

- Dissatisfied
- Very disstisfied

9. I would choose the same treatment again.

- Yes
- No
