# Supplementary material for: Peyronie’s disease in Spain: a prospective study
Source: Sex Med. 2026 May 11;14(4):qfag006. doi: 10.1093/sexmed/qfag006 (PMC13158231; doi:10.1093/sexmed/qfag006)
Supplement: qfag006_Supplemental_Files [file qfag006_supplemental_files.zip › Supplementary_table_1_qfag006.pdf]

Supplementary Table 1. PDQ answers

Items	Value
**Concern about damaging penis while having vaginal intercourse**, median (IQR) (N=213)	1.0 (0.0-2.0)
None, n (%)	75 (35.2)
Mild, n (%)	61 (28.6)
Moderate, n (%)	58 (27.4)
Severe/Very severe, n (%)	19 (8.9)
**Bending or collapsing of penis while having vaginal intercourse**, median (IQR) (N=212)	1.0 (0.0-2.0)
None, n (%)	72 (34.0)
Mild, n (%)	56 (26.4)
Moderate, n (%)	46 (21.7)
Severe/Very severe, n (%)	38 (17.9)
**Trouble inserting erect penis into partner's vagina**, median (IQR) (N=214)	2.0 (1.0-3.0)
None, n (%)	42 (19.6)
Mild, n (%)	51 (23.8)
Moderate, n (%)	63 (29.4)
Severe/Very severe, n (%)	58 (27.1)
**Difficulty with some positions that you used to enjoy when having vaginal intercourse**, median (IQR) (N=213)	2.0 (1.0-3.0)
None, n (%)	24 (11.3)
Mild, n (%)	43 (20.2)
Moderate, n (%)	64 (30.0)
Severe/Very severe, n (%)	82 (38.5)
**Awkwardness with some positions that you used to enjoy when having vaginal intercourse**, median (IQR) (N=214)	2.0 (1.0-3.0)
None, n (%)	22 (10.3)
Mild, n (%)	41 (19.2)
Moderate, n (%)	64 (29.9)
Severe/Very severe, n (%)	77 (36.0)

**Items**	**Value**
**Discomfort with some positions that you used to enjoy when having vaginal intercourse**, median (IQR) (N=211)	2.0 (1.0-3.0)
None, n (%)	35 (16.6)
Mild, n (%)	45 (21.3)
Moderate, n (%)	66 (31.3)
Severe/Very severe, n (%)	65 (30.8)
**In the last 24 hours, how much pain or discomfort have you felt in your penis when it was not erect?**, median (IQR) (N=214)	0.0 (0.0-1.0)
**Thinking about the last time you were erect, how much pain or discomfort did you feel in your penis when it was erect?**, median (IQR) (N=213)	1.0 (0.0-3.0)
**Thinking about the last time you were erect, how much pain or discomfort did you feel in your penis when having vaginal intercourse?**, median (IQR) (N=210)	2.0 (0.0-4.0)
**Thinking about the last time you had an erection, how bothered were you by any pain or discomfort you may have felt in your erect penis?** (N=215)	
Not at all bothered, n (%)	111 (51.6)
A little bit bothered, n (%)	37 (17.2)
Moderately bothered, n (%)	38 (17.7)
Very bothered, n (%)	15 (7.0)
Very/Extremely bothered, n (%)	2 (1.0)
**Thinking about the last time you looked at your erect penis, how bothered were you by the way your penis looked?** (N=213)	
Not at all bothered, n (%)	8 (3.8)
A little bit bothered, n (%)	48 (22.5)
Moderately bothered, n (%)	60 (27.9)
Very bothered, n (%)	69 (32.4)
Very/Extremely bothered, n (%)	28 (13.1)
**Does your Peyronie's Disease make having vaginal intercourse difficult or impossible?** (Yes) (N=214)	98 (45.8)

Items	Value
**Thinking of the last time you had or tried vaginal intercourse, how bothered were you by your Peyronie's Disease?** (N=99)	
Not at all bothered, n (%)	2 (2.0)
A little bit bothered, n (%)	19 (19.2)
Moderately bothered, n (%)	29 (29.3)
Very bothered, n (%)	37 (37.4)
Very/Extremely bothered, n (%)	12 (12.1)
**Are you having vaginal intercourse less often than you used to due to your Peyronie's Disease?** (Yes) (N=211)	144 (68.2)
**How bothered are you with having vaginal intercourse less often?** (N=103)	
Not at all bothered, n (%)	3 (2.9)
A little bit bothered, n (%)	15 (14.6)
Moderately bothered, n (%)	29 (28.2)
Very bothered, n (%)	49 (47.6)
Very/Extremely bothered, n (%)	7 (6.8)

Abbreviations: IQR, interquartile range; PDQ, Peyronie's Disease Questionnaire
