# Supplementary material for: Peyronie’s disease in Spain: a prospective study
Source: Sex Med. 2026 May 11;14(4):qfag006. doi: 10.1093/sexmed/qfag006 (PMC13158231; doi:10.1093/sexmed/qfag006)
Supplement: qfag006_Supplemental_Files [file qfag006_supplemental_files.zip › Supplementary_table_2_qfag006.pdf]

Supplementary Table 2. EIIF-5 answers

Items	n (%)
**How do you rate your confidence that you could get and keep an erection?** (N=420)	
Very low.	39 (9.3)
Low	90 (21.4)
Moderate	108 (25.7)
High	107 (25.5)
Very high	76 (18.1)
**When you had erections with sexual stimulation, how often were your erections hard enough for penetration?** (N=420)	
Almost never or never	43 (10.2)
A few times (much less than half the time)	72 (17.1)
Sometimes (about half the time)	66 (15.7)
Most times (much more than half the time)	95 (22.6)
Almost always or always	144 (34.3)
**During sexual intercourse, how often were you able to maintain your erection after you had penetrated your partner?** (N=418)	
Almost never or never	62 (14.8)
A few times (much less than half the time)	65 (15.6)
Sometimes (about half the time)	69 (16.5)
Most times (much more than half the time)	90 (21.5)
Almost always or always	132 (31.6)
**During sexual intercourse, how difficult was it to maintain your erection to completion of intercourse?** (N=419)	
Extremely difficult	41 (9.8)
Very difficult	59 (14.1)
Difficult	69 (16.5)
Slightly difficult	112 (26.7)
Not difficult	138 (32.9)

Items	n (%)
**When you attempted sexual intercourse, how often was it satisfactory for you?** (N=419)	
Almost never or never	46 (11.0)
A few times (much less than half the time)	72 (17.2)
Sometimes (about half the time)	69 (16.5)
Most times (much more than half the time)	96 (22.9)
Almost always or always	136 (32.5)

IIEF-5, 5-item version of International Index of Erectile Function; N, number of patients with available data; n, patients with event
